# Design and Validation of the Non-Verbal Immediacy Scale (NVIS) for the Evaluation of Non-Verbal Language in University Professors

**DOI:** 10.3390/ijerph19031159

**Published:** 2022-01-20

**Authors:** Pilar Puertas-Molero, Félix Zurita-Ortega, Gabriel González-Valero, José Luis Ortega-Martín

**Affiliations:** 1 Department of Didactics of Musical, Artistic and Corporal Expression, Faculty of Education Sciences, University of Granada, 18071 Granada, Spain; pilarpuertas@correo.ugr.es (P.P.-M.); felixzo@ugr.es (F.Z.-O.); 2 Department of Didactics of Musical, Artistic and Corporal Expression, Faculty of Education and Sport Sciences (Melilla), University of Granada, 52006 Melilla, Spain; 3 Department of Didactics of Language and Literature, Faculty of Education Sciences, University of Granada, 18271 Granada, Spain; ortegam@ugr.es

**Keywords:** non-verbal language, education, validation, professors

## Abstract

Knowledge and awareness of how to use non-verbal language is essential for the educational field. For this reason, the aim of this study was to develop a validation that validly and reliably measures the analysis of non-verbal language in university teachers. Content validation was carried out by applying the Delphi technique and through an exploratory and confirmatory analysis. The validity of understanding is given by the application of the scale to 1316 university teachers between 24 and 67 years of age. The initial data collected through the Delphi technique provided some modifications. The final scale, called Non-verbal immediacy, was composed of a total of 26 items that presented satisfactory adjustments in both comprehension and outcome validity. Confirmatory factor analysis determined three dimensions (kinesics, paralanguage, and proxemics). These factors will be a new element for future lines of research related to the teaching-learning process, as high relationships have been demonstrated between non-verbal language and psychosocial aspects implicit in teaching practice, as well as comprehension and student learning.

## 1. Introduction

Currently, non-verbal language is a competence that professors have to acquire and control, since it has a significant impact on their own work development and especially on the attitude and relationship with students [1,2,3]. Even though it is a characteristic that favors correct job performance, it sometimes goes unnoticed since it acts involuntarily in daily life, generating emotional states in students such as motivation, dissatisfaction, or aversion [4].

In this sense, it is highlighted that the educators’ work consists of making decisions about those methodological aspects that facilitate the acquisition of the objectives by the students, which places greater emphasis on what is or is not said on how it is transmitted [5]. Likewise, Darling and Dannels [6], Nayernia et al. [7], and Yazici and McKenzie [8] stress that communication skills have positive repercussions on school success, emphasizing that it is more important to possess optimal communication skills than handle specific knowledge on the subject.

According to educational institutions, the competences that professors must develop to guarantee students’ success are very broad, which, following the classification of Valdivieso et al. [9], are: the socio-emotional capacity, responsible for ensuring school coexistence; empathy; class dynamization; conflict mediation [10,11,12]; instrumental competence, which involves adequate planning and control of the class and the correct adaptation to new situations [12,13]; and finally, the competence in relational communication, which is of great importance, although sometimes very neglected, and is in charge of controlling what and how messages are transmitted through non-verbal communication [14,15].

The non-verbal communication of educators refers to those behaviors that indicate emotions, increasing the affection of the students towards the teacher, the course itself, and the content taught [16]. Through this, professors establish optimal relationships and promote a positive climate, which has a pleasant impact on interest, effective learning, and the feeling of cohesion and belonging to a group [17,18].

However, teaching tasks are changing because the role of the student in class is increasingly active, which, likewise with the classmates who build their own knowledge, must be directed so that it is meaningful [19]. This gives professors a more mediating character in which they have to offer opportunities and feedback through the use of verbal and non-verbal language simultaneously, motivating and collaborating so that said process develops optimally [20,21,22].

The reality of the communication of the human being is understood in a linear way as opposed to verbal language. However, with words, communication remains incomplete, since all discourse is not strictly verbal, but is characterized by being simultaneous [23]. The nature of our corporeality allows us to generate body postures, gestures, and attitudes that arise parallel to words and are more significant [24]. The teaching styles and strategies refer to the way in which professors transmit knowledge, associating this fact on many occasions with expressive movements [25]. Therefore, non-verbal communication in the educational field is approached in the context of four dimensions: kinesis, proxemics, chronology, and paralanguage [22,26].

For communication to be effective, it is necessary that all dimensions converge consistently; that is, they seek to transmit the same message, strengthening and building it up according to each educational situation [27]. Accordingly, professors who manage to transmit messages while taking care of their verbal and non-verbal communication are later categorized by their students as more credible [28,29]. Non-verbal communication is more effective and immediate than the use of oral language, although due to the lack of awareness of its own use, there are many occasions when contradictory messages are transmitted [30]. Based on the above, the need arises to develop a tool that establishes and analyzes the use of non-verbal language.

Having analyzed the most commonly used instruments for the evaluation of this construct, the teacher interaction questionnaire (TIQ), validated by Chiew-Goh and Fraser [31], which is applied to both professors and students in order to corroborate the vision of both, stands out. Through this instrument, both proxemics and kinesis are measured. In this sense, one of the most complete questionnaires regarding professors’ competencies is the primary education teacher self-perceived competence assessment scale (PETSCAS), which was validated by Valdivieso et al. [9]. This instrument consists of three factors that together comprise teacher training. In the first factor, the variables of coexistence, group identity, affective involvement, communicative adaptability, empathy, awareness, and self-efficacy stand out. 

Within the second factor, the variables of assertiveness, leadership, conflict resolution skills, and non-verbal and paraverbal communication stand out. The third factor is made up of the variables of adaptability to new situations, planning ability, and instructional control.

Likewise, it is necessary to highlight the study by Moreno-Murcia and Huéscar [32], in which they validated the Castilian questionnaire on perceptions of teacher feedback-revised (PTF-R), in which four factors are evaluated: two at the verbal level and the other two on non-verbal language, more specifically, paralanguage.

Given the insufficiency of questionnaires and scales adjusted to Spanish that cover the notion of non-verbal language in the field of education, the validation of a questionnaire is undertaken in a population with its own characteristics, which will provide higher validity rates to research in the field of the teaching profession, as well as the possibility of comparing data with previous studies. Based on the study problem answered, the following research question was posed: Does the validation of the questionnaire provide reliable values for the study of nonverbal language in professors? Thus, the aims pursued are: (a) Study the validity of the content of the questionnaire through the consensus and endorsement of experts using the Delphi technique; (b) Evidence the level of assimilation of this instrument after being applied to a representative number of university professors; (c) Analyze the reliability of the instrument; and (d) Substantiate the complexity of the construct by applying a Confirmatory Factor Analysis (CFA).

## 2. Materials and Methods

### 2.1. Sample

To carry out the assessment and evaluation of the instrument, the Delphi method was used [33], with the collaboration of experts being essential, which is usually quite common and widely used by multiple researchers [34,35]. In response to the postulates proposed by Pozo et al. [36], two groups were formed to validate this instrument: one of them was in charge of coordination and the other was made up of experts. The first group was composed of the participants of this study that present the features of knowledge of the technique, ease of intercommunication [37], and are researchers related to the topic (university professors). In this sense, the group of experts was constituted based on the criteria set forth by Brill et al. [38], who place special emphasis on the relationship each expert has with the subject to be treated, their professional practice, own qualities, and professional background.

In response to this, the experts selected for this research are university researchers and professors of recognized prestige within the field of knowledge that concerns us. It should be noted that the appropriate number should be between 7 and 30 experts. For this study, there were 18 participating specialists—university professors with a doctoral degree and graduates in Physical Education (41.6% of men and 58.4% of women) with an average university teaching experience of *M* = 17.23 ± 3.25 years. Based on the aforesaid, the study proceeded with methodological sequencing, which was structured in three phases: preliminary, exploratory, and final.

In the preliminary phase, the group formed by the coordinators was in charge of delimiting the problem of research. The selection of experts was established (by requesting their commitment and collaboration), and both partial and final results were interpreted, making the necessary adaptations and rectifications.

The instrument proposal was elaborated, as well as its experimental adaptation and its final version, in the exploratory phase. The first version was submitted to be analyzed and discussed by the group of coordinators, who initiated the appropriate adaptations and rectifications by means of the qualitative criteria that presented a greater agreement. This last adaptation was submitted to a second round by the group made up of experts, in order to obtain information on the most stable qualitative and quantitative criteria. For this, the experts were selected, they were invited to participate, and they were provided via email with the instrument, where firstly, on an initial page, they were shown an explanatory introduction on the research topic, together with a record sheet where the data were recorded. Additionally, the objectives of the questionnaire and the method to complete it were explained. The latter is presented on a Likert-type scale with three response options (categorized as high, medium, and low) according to the degree of the adaptation of the item to the dimension to be studied. An open question is also posed to obtain qualitative evaluations of the items raised. Thirty days were given to respond, and during that month, people were checked on, the completed scales were collected, and the information was analyzed by the leading group.

In the closing phase, the outcomes of the entire validation process of the final version of the questionnaire were synthesized for subsequent application to 1316 university professors in Spain, with an average age of *M* = 45.64 ± 10.33 years, of whom 623 (47.3%) were men and 693 (52.7%) were women. Stratified random sampling techniques were used. Based on the universe of the sample (99,458 university teachers), a sampling error of 0.03 and a confidence interval of 95.5% were established. The inclusion criteria included teachers with doctoral degrees who were teaching at university stage. On this condition, a total of 87 questionnaires were eliminated on the basis of incorrectly completed answers. The study was conducted according to the guidelines of the Declaration of Helsinki and approved by the Board Research Ethics Committee of the University of Granada (1230/CEIH/2020).

### 2.2. Procedure

#### 2.2.1. Instrument Construction

After analyzing the shortcomings of the questionnaires and instruments that were available, it was decided to develop the non-verbal immediacy scale. To do this, the requirements established by Ramos et al. [39] in their research were followed, which respond to: (a) briefness of the items; (b) easiness in its application; (c) simple vocabulary and adapted to the characteristics of the sample; (d) short and closed questions; € attractive in its design and with theoretical support.

#### 2.2.2. General Steps for the Elaboration of the Non-Verbal Immediacy Scale

The scale has been constructed and elaborated from the conditions of a psychological evaluation instrument proposed by Cronbach [40]. The content has been determined through the bibliographic review and the opinion of the experts [41], according to the established recommendations, and will be made through closed questions and five response options.

#### 2.2.3. Elaboration of the Non-Verbal Immediacy Scale

Starting from an elementary set of items that came from various questionnaires and scales related to both the use of non-verbal language and its dimensions that are closely related to the concept developed, the coordinating group prepared an initial experimental version, eliminating some items and dimensions that were misleading and that caused some complexity in the overall understanding of the scale.

The following parameters were used: never, rarely, occasionally, often, and very often. Items were read and grouped into the dimensions: kinesis, paralanguage, and proxemics. The choice was made according to its suitability by a rational criterion, obtaining a total of 26 items that are the foundation for the elaboration of the scale in its first version. These questions came from different sources; some were obtained strictly from the instruments of origin, others were redefined, and others were written specially for this test. The dimensions were altered when distributing the questions and the option was closed from 1 to 5.

#### 2.2.4. Instrument Content Validity

To carry out the study of the validity of the questionnaire, definitions were established of the validity of the content and the extent to which a test adequately represents what has been done [42]. The technique of experts was used to achieve the optimal levels of content validity, and a pilot study was established to determine the comprehension validity of the subjects under study. The experts carried out the assessment of the initial information, the questions, and the general assessment of each one, considering the level of understanding and/or adequacy of the writing.

With regard to the items, a set of statistical indicators have been considered, such as the discrimination index and the descriptive statistics. In order to give the data adequate accuracy, it was found necessary to complete a study of the reliability and validity, and the latter would go through the fulfillment of psychometric requirements with a sufficient Cronbach reliability coefficient and confirmatory factor analysis [40,43]. For the verification of all this, the statistical programs SPSS 24.0, FACTOR Analysis 9.3.1, and AMOS were used.

#### 2.2.5. Instrument Validity

To control the validity, a pilot study was implemented where, after being applied to 1316 university professors (with a maximum time of 5 to 6 min), the degree of understanding was established from a qualitative viewpoint, registering the doubts and suggestions that were perceived in the questionnaire.

### 2.3. Data Analysis

For the analysis of qualitative data, content analysis has been used; quantitative data, analysis of descriptive statistics and estimation of internal consistency have been carried out with the SPSS 24.0 program, the EFA (Exploratory Factor Analysis) was done with FACTOR Analysis 9.3.1, and the CFA was done with AMOS. Both analyses were conducted for the total sample of 1361 university teachers with a mean age of *M* = 45.64 ± 10.33 years.

First, an analysis of the distribution of the items was carried out by means of asymmetry and kurtosis to identify possible distortions that could influence the results, including values between ±2 [44,45]. Furthermore, multicollinearity analysis was carried out among the items in order to estimate the existence of any redundant variables (inter-item correlations greater than 0.95). Secondly, EFA was performed to identify the item structure by means of the correlation matrix. The Kaiser–Meyer–Olkin coefficient (KMO) was also examined to compare the correlations between variables by identifying common factors. Its values range between 0 and 1, with values above 0.80 being indicators that pairs of variables can be explained by other variables [46]. In addition, Bartlett’s sphericity test was applied to test the null hypothesis that the correlation matrix is equal to the identity matrix and, therefore, the correlations between the variables are 0. On the other hand, in order to identify whether an item belongs to a factor, the factorial load was established as a criterion that the factorial load is equal to or greater than 0.40 [47]. As the oblique rotation method was applied, the correlations between factors were analyzed with statistical significance and the magnitude of the effect followed Cohen’s criteria, with the effects being: small (*r* ≥ 0.10; *r*^2^ ≥ 0.01), medium (*r* ≥ 0.30, *r*^2^ ≥ 0.09), and large (*r* ≥ 0.50, *r*^2^ ≥ 0.25). 

Thirdly, the internal consistency of each of the factors was analyzed using Cronbach’s alpha coefficient. Fourthly, confirmatory factor analysis was carried out. To assess the fit of the model, goodness-of-fit indices were applied. The following goodness-of-fit indicators were applied: CFI, comparative fit index; GFI, goodness-of-fit index; RMR, root mean square root; and RMSEA, root mean square root of approximation. An acceptable fit is considered if the GFI values are close to 0.90, RMR < 0.08, and RMSEA < 0.06 [48]. 

Finally, the CFA was performed to corroborate the belonging of each item to the dimensions found in the EFA. Once the multidimensionality of the instrument was corroborated, it was applied independently to each of the factors of NVIS. The Multilog and Parscale analyses [49] were used to estimate the model parameters using the marginal maximum likelihood method. For each item grouping, the loading value of the first factor was identified in order to establish whether there is a dominant factor [50]. The discrimination ability of the items was then assessed by means of the corrected item-test correlations, which should be greater than 0.20 (*p* < 0.05), as proposed by Kline [51]. Moreover, item discrimination values were calculated and the parameters [52] were estimated and their respective errors (Ee) were reported. In order to assess the goodness of fit to NVIS, the invariance of the parameters was analyzed through the chi-square (χ2) test, which shows that items present results that are not statistically significant (*p* > 0.05). Finally, marginal reliability was calculated in order to establish whether the scores obtained were reliable.

## 3. Results

The data for the findings regarding the content validity of the instrument were obtained by means of qualitative techniques and processed through content analysis in order to collect evidence regarding the conceptual, cultural, and linguistic validity of the Scale of Non-Verbal Immediacy. The qualitative contributions are completed with the quantitative contributions provided by the experts for each item. The integration of the contributions of the two groups that have constituted two separate sources guarantees the adequacy of the instrument.

Of the 26 items that compose the questionnaire, 17 of them do not suffer modification, as the expert evaluations are close to the score 3 and in none of them is any alternative proposed; furthermore, the remaining 9 with values near 2 are adjusted following the contributions and opinions of the group. The final formulation is agreed upon with the coordinating group.

SPSS 24.0 and FACTOR Analysis 9.3.1 were used for the exploratory factor structure. The descriptive values of the study were studied in the first part of the analysis of the results, following the steps recommended by the experts [53], not disregarding any item since there are no figures greater than 2.00 in the dispersion tests (asymmetry and kurtosis), as shown in Table 1.

Subsequently, by using the FACTOR Analysis program [54], as can be seen in Table 2, three factors have been rotated for the pilot test. The Bartlett statistic, [14,886.0 (*df* = 325; *p* = 0.000)], and the KMO = 0.873, used for testing if the sample comes from populations with the same variance and if it presents a good sample adequacy, indicate a good fit of the data to be submitted to factor analysis. The three factors extracted explain 53.1% of the total variance: the CFI was 0.957, the Goodness of Fit Index (GFI) was 0.967, the Adjusted Goodness of fit Index (AGFI) also obtained 0.957, and the root mean square of the residuals (RMSR) was 0.058. All of the data indicate an outstanding fit for these items. For the reliability analysis, the Cronbach’s Alpha with a value of 0.867 for the general scale and over 0.700 in the three factors extracted. Variables V15 and V25 have been suppressed, due to not loading properly (values that do not exceed 0.300 and difference between the two superiors less than 0.100).

**Figure 1:**
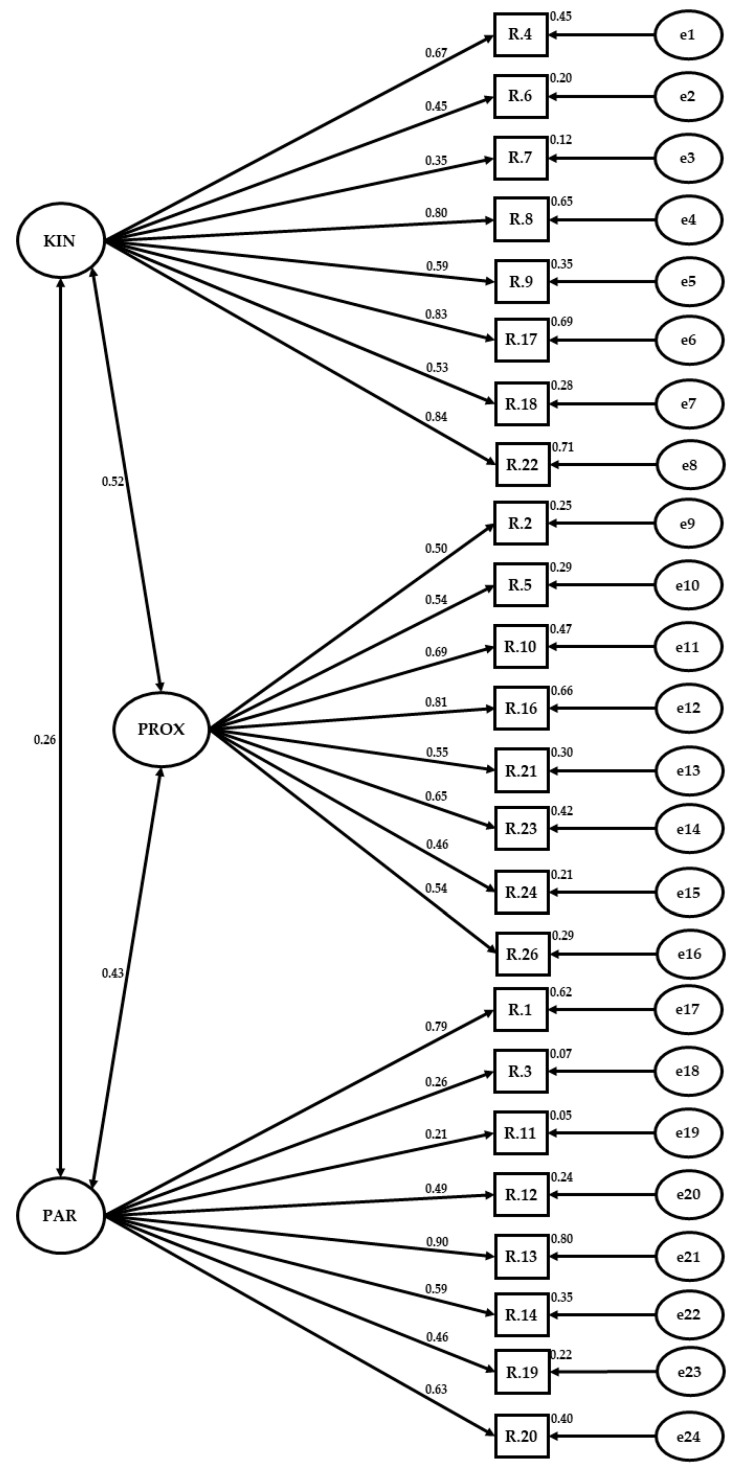
Verifying factor analysis of the Non-Verbal Immediacy Scale. Factor 1: Kinesis (KIN), Factor 2: Proxemic (PROX), Factor 3: Paralanguage (PAR). Note: Two-way effect between variables (↔); One-way effect between variables (←); Variables (R); measurement error (e).

Once verified by means of the EFA and the reliability of the items, the validity of the instrument uses the CFA, for which the 24 selected questions were classified into a previous theoretical structure of three elements: kinesis, proxemics, and paralanguage, previously confirmed in the exploratory analysis. Eventually, the factorial structure has been analyzed using a CFA where three factors are considered. This provision is a priori to what states that the results of the model are entirely confirmatory. As what happened with the EFA, the indices show a reasonably adequate adjustment of the proposed model. In this way, the CFI index gets a value of 0.905 and the TLI is 0.932. The chi-square sets a value of 4352.647 with 249 degrees of freedom. Finally, the RMSEA estimates the model as a good fit, with an index of 0.071. In this way, and relying jointly on all the indicated indices (Figure 1), it can be verified that the model that has been proposed presents a valid and satisfactory approximation to the data and can contribute to sustaining the hypothesis of the multidimensionality of the construct.

Therefore, an analytical review of the proposed factor structure is finally made, in a manner that the estimates of the factor saturations for each of the items in their respective factors, as shown in Figure 1.

## 4. Discussion and Conclusions

The objective of this research has been to analyze and validate the content of the Non-Verbal Immediacy Scale in a sample of participants from university professors. Regarding the results obtained, it is worth mentioning that they show the satisfactory metric quality of the instrument when evaluated through confirmatory analysis. Similarly, it should be noted that they have demonstrated an adequate fit to the proposed model. In summary, it should be highlighted that the results indicate the appearance of three factors: kinesis, paralanguage, and proxemics.

The phases recommended by the general scientific literature were followed during design and validation [33,55,56]. Likewise, it is highlighted that the group of participating experts who contributed to the instrument validation (*n* = 18) present quality criteria, the number of which are higher than those who have participated in similar research, more specifically in the study prepared by Chiang-Salgado et al. [57] involving a total of 10 judges and in the study by Ceballos-Vásquez et al. [58], in which 14 experts participated.

In the same way, adequate reliability can be seen around the internal consistency, both for the questionnaire in general and in the three dimensions that facilitate its application to any educational scope. The psychometric results extracted from the factorial structure and reliability enhance the aspects of content validity and show good psychometric quality.

The three dimensions found allow us to assess the use of non-verbal language, since it is unconscious and immediate in society [59]. In this sense, non-verbal aspects play a fundamental role in the professional field of professors since they have a very important role in building relationships and in how social control is manifested and perceived. Although, as shown by authors such as Aspelin [12] and Kell and Swet [27], these social relationships can also manifest as negative social sanctions, which are fragile and their consequences are very unpredictable, due to the attempt of educators to make students follow a rigid order and a pattern of behaviors. Little control of this competence can generate unexpected negative situations, hence demonstrating the importance of the teacher having control of non-verbal language [30].

The final form of the questionnaire includes three dimensions, as well as an additional general Non-Verbal Immediacy Index, which is established by adding the 26 items that make up the scale. The data obtained establish new study viewpoints on the importance of non-verbal language in professors, since it has great power over sociability and professional satisfaction [60].

Therefore, this scale should be considered as a tool that allows university professors to know if their performance is being adequate, as well as to promote educational quality and teacher well-being [61]. The Nonverbal Immediacy scale supposes empirical evidence as a tool that can be of help for educators to recognize and adapt their professional action.

This study was not exempt from limitations, as the sample analyzed is centered on a group of university professors from different areas of knowledge and with rather heterogeneous ages. As such, this is a construct that has been rarely studied, as the systematic analysis of the scientific literature in the main high-impact databases demonstrates. In addition, stratified random sampling techniques can generate debate among readers, just as how the EFA and CFA were conducted on the same sample. However, these factors will be a new element for future lines of research related to the teaching-learning process, as high relationships have been demonstrated between non-verbal language and psychosocial aspects implicit in teaching practice, as well as student understanding and learning. In this way, this instrument can be used as a tool for self-evaluation and heteroevaluation in the educational field. Finally, the scientific community is encouraged to use and validate this instrument in teachers of other educational stages.

## Figures and Tables

**Table 1:** Descriptives of the Non-Verbal Immediacy Scale.

Scale Items	*M*	*SD*	*V*	*A*	*K*
L1. I use my hands and arms to gesture as I speak	4.17	0.988	0.977	−1.021	0.208
L2. I touch others on the shoulder or arm when I speak to them	2.63	1.340	1.796	0.273	−1.167
L3. I use a monotonous or soft voice when speaking	3.72	1.098	1.206	−0.576	−0.427
L4. I look up or away when I talk to other people	4.20	1.027	1.056	−1.264	0.899
L5. I avoid others when they touch me as we speak	3.94	1.184	1.402	−0.962	−0.019
L6. I have relaxed posture as we speak	3.77	1.040	1.083	−0.592	−0.320
L7. I frown when talking to others	3.86	1.008	1.016	−0.681	−0.062
L8. I avoid eye contact while talking to someone	4.43	0.889	0.792	−1.731	1.742
L9. I have tense posture when I talk to people	4.15	0.898	0.807	−1.074	1.023
L10. I stay close to the person I am talking to (sitting or standing)	3.74	1.043	1.089	−0.557	−0.320
L11. My voice is monotonous or soft when I speak to people	3.54	1.083	1.174	−0.385	−0.514
L12. I use a variety of vocal expressions when talking to people	3.89	0.875	0.766	−0.496	−0.110
L13. I gesture as I speak	4.00	1.004	1.009	−0.786	−0.137
L14. I am encouraged when I speak	4.09	0.789	0.624	−0.621	0.210
L15. I have a dull or bland facial expression when talking to others	4.22	0.838	0.704	−0.981	0.704
L16. I get close to people when I talk to them	3.69	1.019	1.038	−0.565	−0.171
L17. I look directly at the person I am talking to	4.31	0.869	0.756	−1.282	1.324
L18. I stay rigid when I talk to others	4.02	0.876	0.768	−0.733	0.193
L19. I vary the tone of my voice frequently when speaking to others	3.72	1.040	1.082	−0.589	−0.256
L20. I avoid gesturing when I speak	4.32	0.915	0.838	−1.450	1.767
L21. I lean towards the person I’m talking to	2.90	1.160	1.347	0.019	−0.799
L22. I maintain eye contact with the person I am talking to	4.27	0.886	0.786	−1.239	1.228
L23. I don’t stay close to the people I talk to (sitting or standing)	4.01	1.013	1.027	−0.929	0.343
L24. I don’t face my body towards the person I’m talking to	4.01	1.120	1.256	−1.043	0.243
L25. I smile when I talk to someone else.	4.15	0.833	0.694	−0.779	0.329
L26. I avoid touching people when I talk to them.	3.22	1.325	1.757	−0.315	−1.024

Note: Median (*M*); Standard Deviation (*SD*); Variance (*V*); Asymmetry (*A*); Kurtosis (*K*).

**Table 2:** Factor load of the dimensions of the Non-Verbal Immediacy Scale.

Variables	F1	F2	F3	Variables	F1	F2	F3
V01. I use my hands and arms to gesture as I speak	−0.351	0.114	0.846	V04	0.804		
V02. I touch others on the shoulder or arm when I speak to them	−0.366	0.757	0.128	V06	0.539		
V03. I use a monotonous or soft voice when speaking	0.174	−0.302	0.496	V07	0.627		
V04. I look up or away when I talk to other people	0.804	−0.090	−0.047	V08	0.837		
V05. I avoid others when they touch me as we speak	0.279	0.550	−0.162	V09	0.814		
V06. I have relaxed posture as we speak	0.539	0.016	0.013	V17	0.662		
V07. I frown when talking to others	0.627	−0.052	−0.199	V18	0.643		
V08. I avoid eye contact while talking to someone	0.837	0.019	−0.012	V22	0.662		
V09. I have tense posture when I talk to people	0.814	−0.001	−0.097	V02		0.757	
V10. I stay close to the person I am talking to (sitting or standing)	0.155	0.532	0.081	V05		0.550	
V11. My voice is monotonous or soft when I speak to people	0.109	−0.226	0.424	V10		0.532	
V12. I use a variety of vocal expressions when talking to people	0.105	−0.027	0.582	V16		0.753	
V13. I gesture as I speak	−0.357	0.079	0.978	V21		0.632	
V14. I am encouraged when I speak	0.160	0.055	0.638	V23		0.564	
V15. I have a dull or bland facial expression when talking to others	0.390	0.006	0.366	V24		0.366	
V16. I get close to people when I talk to them	0.053	0.753	0.060	V26		0.800	
V17. I look directly at the person I am talking to	0.662	0.052	0.209	V01			0.846
V18. I stay rigid when I talk to others	0.643	0.108	−0.013	V03			0.496
V19. I vary the tone of my voice frequently when speaking to others	−0.068	−0.012	0.648	V11			0.424
V20. I avoid gesturing when I speak	−0.024	0.121	0.602	V12			0.582
V21. I lean towards the person I’m talking to	−0.201	0.632	0.117	V13			0.978
V22. I maintain eye contact with the person I am talking to	0.662	0.042	0.198	V14			0.638
V23. I don’t stay close to the people I talk to (sitting or standing)	0.286	0.564	−0.062	V19			0.648
V24. I don’t face my body towards the person I’m talking to	0.251	0.366	0.020	V20			0.602
V25. I smile when I talk to someone else.	0.251	0.187	0.266				
V26. I avoid touching people when I talk to them.	−0.091	0.800	−0.158				
				α = 0.867	α = 0.849	α = 0.810	α = 0.787

## Data Availability

The data that support the findings of this study are available from the corresponding author, upon reasonable request.
